# Functional and oncological outcomes of patients with proximal humerus osteosarcoma managed by limb salvage

**DOI:** 10.1186/s10195-024-00756-4

**Published:** 2024-04-18

**Authors:** Wael Mohamed Safwat Sadek, Ibrahim Khairy Fayed Elshamly, Moustafa Saladin Mohammed Salem, Wessam Gamal AbouSenna, Emad Ebeid, Walid Atef Ebeid

**Affiliations:** 1 Department of Orthopedics and Traumatology, Cairo University, Cairo, Egypt; 2 Department of Paediatric Oncology and Haematology, National Cancer Institute, Cairo University, Cairo, Egypt

**Keywords:** proximal humerus, osteosarcoma, limb salvage, outcome

## Abstract

**Background:**

Osteosarcoma is the most common primary bone malignancy in skeletally immature patients. The proximal humerus is the third most common site of osteosarcoma. The literature shows a paucity of published data concerning the outcome of proximal humerus osteosarcoma managed by limb salvage. The purpose of this study was to answer the following questions: (1) do patients with proximal humerus osteosarcoma managed by limb salvage and neoadjuvant chemotherapy show good functional and oncological outcomes, and (2) are there any prognostic factors that are associated with better oncological and functional outcomes?

**Materials and methods:**

The study was a retrospective case series study assessing the overall outcome of 34 patients with proximal humerus osteosarcoma. Eighteen patients were males (53%) while 16 were females. Biological reconstruction was done in 15 patients (44%), while nonbiological reconstruction was done in 19 patients. Resections were mainly intraarticular (82%). Functional outcome was assessed using the Musculoskeletal Tumor Society (MSTS) score, while oncological outcome was assessed based on local recurrence and development of chest metastasis. Comparisons between quantitative variables were done using the nonparametric Mann–Whitney test. To compare categorical data, the chi-square (*χ*^2^) test was performed. The exact test was used instead when the expected frequency was less than 5. Correlations between quantitative variables were examined using the Spearman correlation coefficient.

**Results:**

The mean MSTS score was 25.5 (range 23–29). A younger age was statistically correlated with a poorer MSTS score (*P* = 0.0016). Six patients out of 34 (17.6%) had local recurrence and four of them (67%) were treated by forequarter amputation. 41% of patients developed chest metastasis, and the majority of them were treated by chemotherapy (71%). In comparison with patients with osteosarcoma at other sites who were also managed in our institution, proximal humerus osteosarcoma patients showed higher incidence rates of local recurrence and chest metastasis along with lower 5-year patient and limb survivorships compared to distal femur, proximal tibia and proximal femur osteosarcoma patients.

**Conclusion:**

Treatment of osteosarcoma of the proximal humerus by limb salvage and chemotherapy yields a good functional outcome. The method of reconstruction does not impact the resultant function. The 5-year survivorship of these patients is 65%. Younger patients have a better oncological outcome and an inferior functional outcome.

**Level of evidence:**

Level IV therapeutic study.

## Introduction

Osteosarcoma is the most common primary bone malignancy in skeletally immature patients. Osteosarcoma commonly occurs in the metaphysis of a long bone. The most common sites are the femur (42%), the tibia (19%) and the humerus (10%). The overall 5-year survival rate for osteosarcoma is 68%, and there is no significant gender difference [17].

Before 1970, most patients with high-grade sarcomas arising in the proximal humerus were treated with forequarter amputation. The development of effective induction and adjuvant chemotherapy protocols prompted Marcove et al. to extend the indications for limb-sparing shoulder girdle resections to include high-grade sarcomas of the proximal humerus and scapula [1].

The optimum method of reconstructing the shoulder after resection of the proximal humerus remains controversial. Options include the use of a fibular or autoclaved humeral autograft, an osteoarticular allograft, an intercalary allograft prosthesis composite, the clavicula pro humero procedure, a 3D-printed custom-made prosthesis or an endoprosthesis. The decision depends on the site and the size of the tumor, the level of resection required to obtain wide, clear margins, the resources available and the abilities of the surgeon.

Local recurrence remains a significant problem after the resection of malignant tumors around the shoulder. This is largely due to the proximity of the neurovascular bundles to the bone, as only marginal margins may be achieved when there is a significant soft-tissue component of the tumor. Moreover, tumors close to the axial skeleton may have a higher incidence of systemic relapse [14].

The aim of this study was to assess the functional and oncological outcomes of patients with proximal humerus osteosarcoma managed by limb salvage and the prognostic factors that affect them.

Since the prevalence of osteosarcoma is low and the proximal humerus is not a common site, assessing the oncologic outcome of a tumor in this location requires many years. Accordingly, we opted for a retrospective analysis of our prospective database. Moreover, the literature shows a paucity of published data concerning the subject of this study. We believe that this study, with its relatively large number of patients with a long follow-up, will be a valuable addition to the literature.

## Materials and methods

The study was a retrospective case series study assessing the overall outcome of 34 patients with proximal humerus osteosarcoma. Eighty-four patients diagnosed with proximal humerus osteosarcoma were managed in our institution from April 1995 to September 2021. Thirty-four of them met the inclusion criteria and were therefore selected**.** Our inclusion criteria included all patients with high-grade conventional osteosarcoma of the proximal humerus who received the full chemotherapy protocol and were managed by limb salvage. Our study included only patients with at least 2 years of follow-up. All patients signed an informed consent; for patients under 18 years of age, the informed consent was signed by their parents or legal guardians.

General information, including demographics, reconstruction type and functional outcomes, were reviewed. Patients included in the study were all diagnosed with conventional high-grade osteosarcoma. 30% of patients had a pathological fracture on presentation. The tumor necrosis and surgical margins were noted in the postoperative pathology notes. All patients received neo-adjuvant and adjuvant chemotherapy in accordance with the EURAMOS (European and American Osteosarcoma Studies) protocol.

All our patients underwent en block resection, with wide and marginal margins obtained. Fifteen patients underwent biological reconstruction with a vascularized fibular graft and shoulder fusion, while the remaining underwent nonbiological reconstruction through the use of a PMMA (polymethyl methacrylate) spacer or endoprosthesis.

Follow-up visits were scheduled every 6 weeks in the first year, every 3 months during the second year and every 6 months thereafter. Local recurrence and distant metastasis were assessed clinically and aided by MRI and CT scan and biopsy when applicable. The Musculoskeletal Tumor Society (MSTS) score [10] was used to evaluate the functional scores of all patients.

Data were coded and entered using the Statistical Package for the Social Sciences (SPSS) version 26 (IBM Corp., Armonk, NY, USA). Quantitative data were summarized using the mean, standard deviation, median, minimum and maximum and categorical data were summarized using frequency (count) and relative frequency (percentage) [3]. Comparisons between quantitative variables were done using the nonparametric Mann–Whitney test. To compare categorical data, the chi-square (*χ*^2^) test was performed. The exact test was used instead when the expected frequency was less than 5 [4]. Correlations between quantitative variables were examined using the Spearman correlation coefficient [5]. *P* values of less than 0.05 were considered statistically significant.

## Results

**Table 1:** Overall description of the study population

	Count	%
Sex		
Male	18	53
Female	16	47
Method of reconstruction		
Biological	15	44
Nonbiological	19	56
Resection type		
Intraarticular	28	82
Extraarticular	6	18
Length resected		
< 15 cm	12	35
> 15 cm	22	65
Deltoid resection		
Yes	22	65
None	12	35
Complications		
Radial nerve neurotmesis	2	6
No	32	94
Treatment of complications		
Tendon transfer	2	100
Construct failure		
Yes	6	17
No	28	83
Management of the failure		
Revision	4	62
VFG	1	25
Open reduction	1	13
Surgical margin		
Wide	29	85
Marginal	5	15
Pathological fracture on presentation		
Yes	10	30
No	24	70
Chemotherapy		
Yes	34	100
Local recurrence		
Yes	6	18
No	28	82
Treatment of local recurrence		
Forequarter amputation	4	67
No	2	33
Chest metastasis		
Yes	14	41
No	20	59
Treatment of chest metastasis		
Chemotherapy + metastasectomy	4	29
Chemotherapy only	10	71
Status at 5-year follow-up		
Alive	22	65
Deceased	12	35

Our population study was young, with a mean age of 15 years. The youngest patient was 3 years old while the eldest was 28 years old. Eighteen patients were males (53%), while 16 were females (Table 1). Biological reconstruction was done in 15 patients (44%), while nonbiological reconstruction was done in 19 patients. Resections were mainly intraarticular (82%). Resection length was more than 15 cm (distal to deltoid insertion) in 65% of patients. Regarding tumor necrosis, a good response (> 90%) was present in 19 patients (56%).

### Functional outcome

**Fig. 1:**
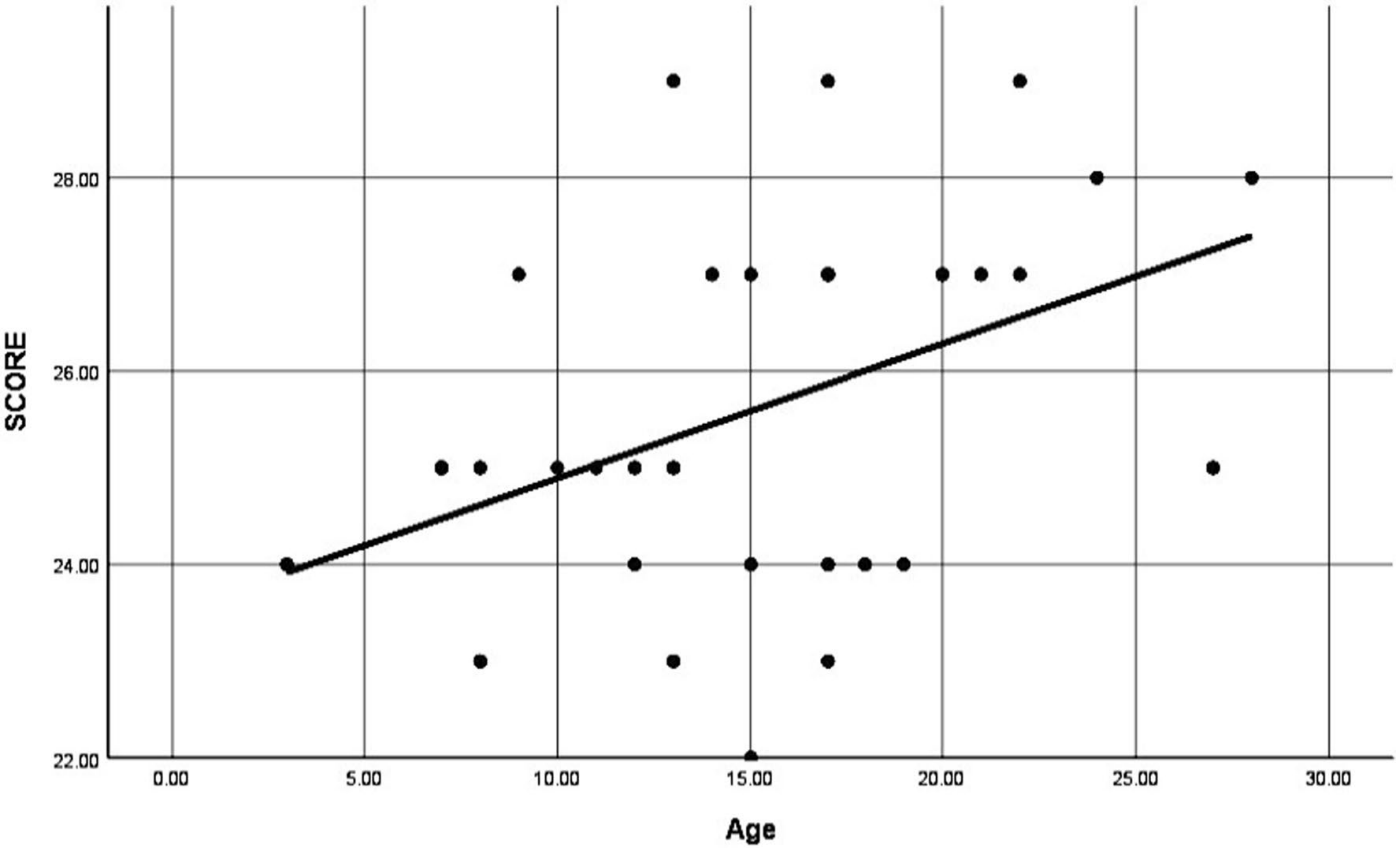
Plot highlighting the direct correlation of a younger age (in years) with a lower MSTS score

The mean MSTS score was 25.5 (range 23–29). A younger age was statistically correlated with a poorer MSTS score (*p* = 0.0016) (Fig. 1). None of the other variables, including resection length, deltoid resection, surgical margins, the method of reconstruction (biological and nonbiological) and construct failure, were correlated with MSTS scores (Table 2).

**Table 2:** Correlations between MSTS score and different variables

	MSTS score					*P* value
	Mean	SD	Median	Minimum	Maximum	
Sex						
Male	25.11	2.08	24.50	22.00	29.00	0.109
Female	26.06	1.65	26.00	23.00	29.00	
Method of reconstruction						
Biological	25.53	1.73	25.00	23.00	29.00	1
Nonbiological	25.58	2.12	25.00	22.00	29.00	
Resection type						
Intraarticular	25.43	1.97	25.00	22.00	29.00	0.439
Extraarticular	26.17	1.72	27.00	24.00	28.00	
Length resected						
< 15 cm	25.25	1.86	25.00	23.00	29.00	0.444
> 15 cm	25.73	1.98	25.00	22.00	29.00	
Deltoid resection						
Yes	25.41	1.79	25.00	23.00	29.00	0.511
No	25.83	2.21	27.00	22.00	29.00	
Complications						
Radial nerve neurotmesis	23.50	0.71	23.50	23.00	24.00	0.107
No	25.69	1.91	25.00	22.00	29.00	
Construct failure						
Yes	24.75	1.91	24.50	23.00	29.00	0.164
No	25.81	1.90	26.00	22.00	29.00	
Surgical margin						
Wide	25.48	1.94	25.00	22.00	29.00	0.603
Marginal	26.00	2.00	25.00	24.00	29.00	
Pathological fracture on presentation						
Yes	25.80	2.04	25.00	23.00	29.00	0.642
No	25.46	1.91	25.00	22.00	29.00	

### Oncological outcome

Six patients (17.6%) had local recurrence and four of them (67%) were treated by forequarter amputation. Local recurrence was not found to be related to any variable—notably the surgical margins (*P* = 0.2), pathological fracture on presentation (*P* = 0.3) and resected length (*P* = 1) (Table 3).

**Table 3:** Local recurrence evaluation

	Local recurrence				*P* value
	Yes		No		
	Count	%	Count	%	
Sex					
Male	3	16.7	15	83.3	1
Female	3	18.8	13	81.3	
Method of reconstruction					
Biological	2	13.3	13	86.7	0.672
Nonbiological	4	21.1	15	78.9	
Resection type					
Intraarticular	4	14.3	24	85.7	0.281
Extraarticular	2	33.3	4	66.7	
Length resected					
< 15 cm	2	16.7	10	83.3	1
> 15 cm	4	18.2	18	81.8	
Deltoid resection					
Yes	3	13.6	19	86.4	0.641
No	3	25.0	9	75.0	
Complications					
Radial nerve neurotmesis	0	0.0	2	100.0	1
No	6	18.8	26	81.3	
Construct failure					
Yes	0	0.0	8	100.0	0.297
No	6	23.1	20	76.9	
Surgical margin					
Wide	4	13.8	25	86.2	0.205
Marginal	2	40.0	3	60.0	
Pathological fracture on presentation					
Yes	3	30.0	7	70.0	0.328
No	3	12.5	21	87.5	

Fourteen patients (41%) developed chest metastasis, and the majority of them were treated by chemotherapy (71%). The most noticeable variable is deltoid resection, which showed a significant inverse correlation with distant metastatic spread (*P* = 0.05). The surgical margins (*P* = 0.6), resected length (*P* = 0.2) and pathological fracture on presentation (*P* = 0.4), among others, were not related to the risk of developing lung metastasis.

### Survivorship

The 5-year survivorship of proximal humeral osteosarcoma in this study was 65%. A younger age was associated with a greater survivorship, with the patients who were deceased at 5 years of follow-up having a mean age of 17 years whereas those who were alive at 5 years of follow-up had a mean age of 13 years (*P* = 0.03) (Table 4). Other factors such as sex, resection length, deltoid resection and the presence of a pathological fracture did not impact survivorship. Survivorship was significantly dependent on an absence of local recurrence (*P* = 0.014) and definitely improved with an absence of chest metastasis (*P* < 0.001).

**Table 4:** Relation between age and survivorship

	Patients who were deceased at 5 years of follow-up					Patients who were alive at 5 years of follow-up					*P* value
	Mean	SD	Median	Minimum	Maximum	Mean	SD	Median	Minimum	Maximum	
Age (years)	17.67	5.25	17.00	8.00	27.00	13.23	5.92	12.50	3.00	28.00	0.034

**Fig. 2:**
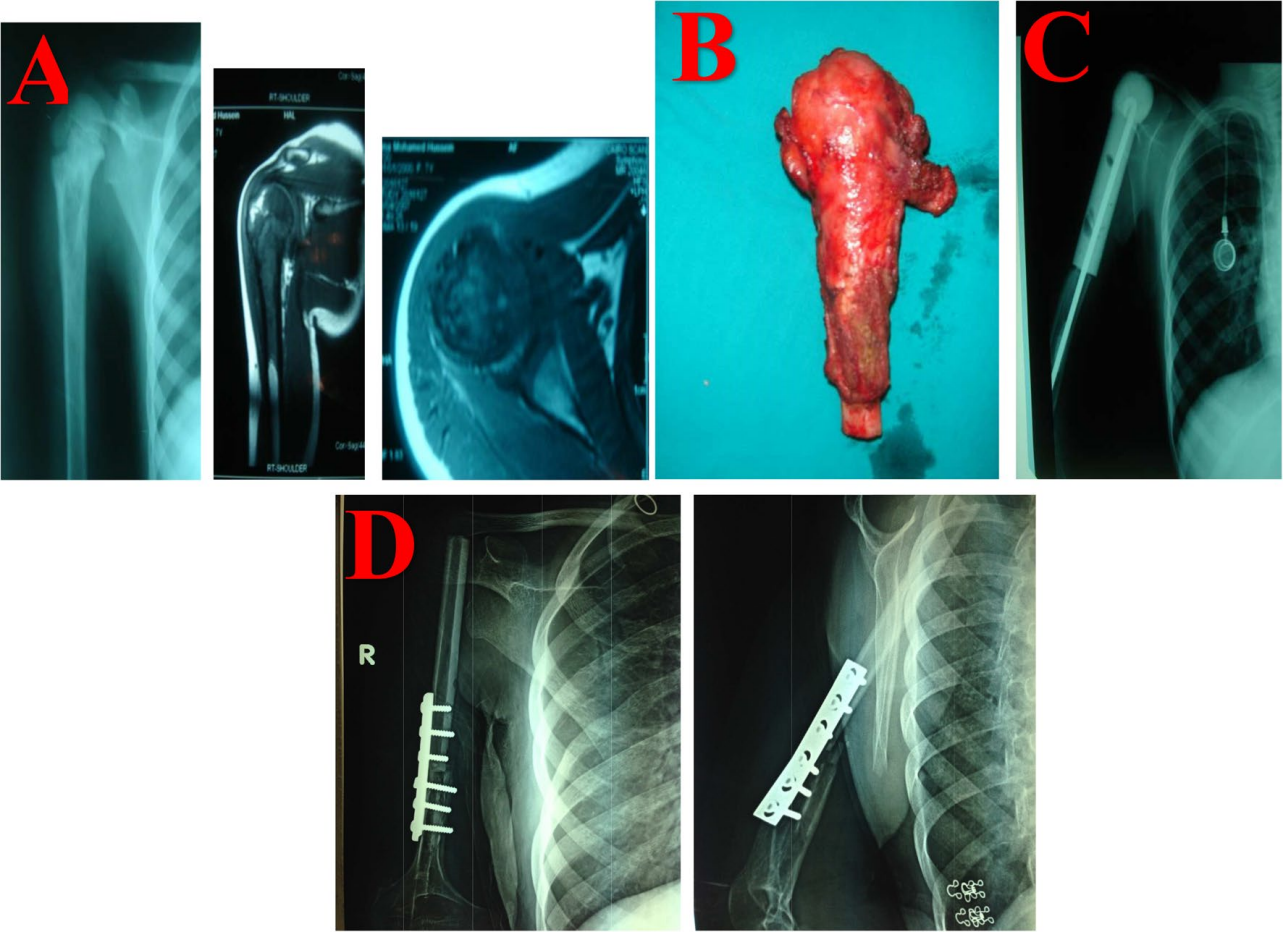
**A** Preoperative investigations, including a shoulder X-ray of the Rt (right) shoulder and MRI with contrast. Note the osteolytic superimposition on the periosteal reaction at the right proximal humerus radiograph. MRI highlighted an abnormal signal intensity on coronal and axial views from a T2-weighted gadolinum contrast study. **B** Resected specimen. **C** Postoperative radiograph in which a PMMA spacer was used. **D** At 7 years of follow-up (in 2014), the patient presented construct failure, as the spacer was exposed, so spacer removal and NVFG (non-vascularized fibular graft) were performed

There was a failure of the reconstructive modality that required revision in 6 patients. The construct survivorship was 82% (Fig. 2). The limb survivorship in our study was 88%, as only 4 patients underwent amputation to treat local recurrence.

### Complications

Radial nerve injury was only encountered in 2 patients (6%), and those injuries were managed later on by tendon transfer. Two patients developed nonunion and were treated by bone grafting. Two patients had proximal migration of the endoprosthesis (MSTS 24) and one had inferior sublaxation (MSTS 25). No further treatment was done for those complications as they did not impact the patients’ functional outcomes and surgical interference would not have improved the range of motion of the shoulder. One patient had skin sloughing and a superficial infection that was managed conservatively. Two PMMA spacers broke and were revised with a more durable PMMA spacer using a humeral nail.

## Discussion

**Fig. 3:**
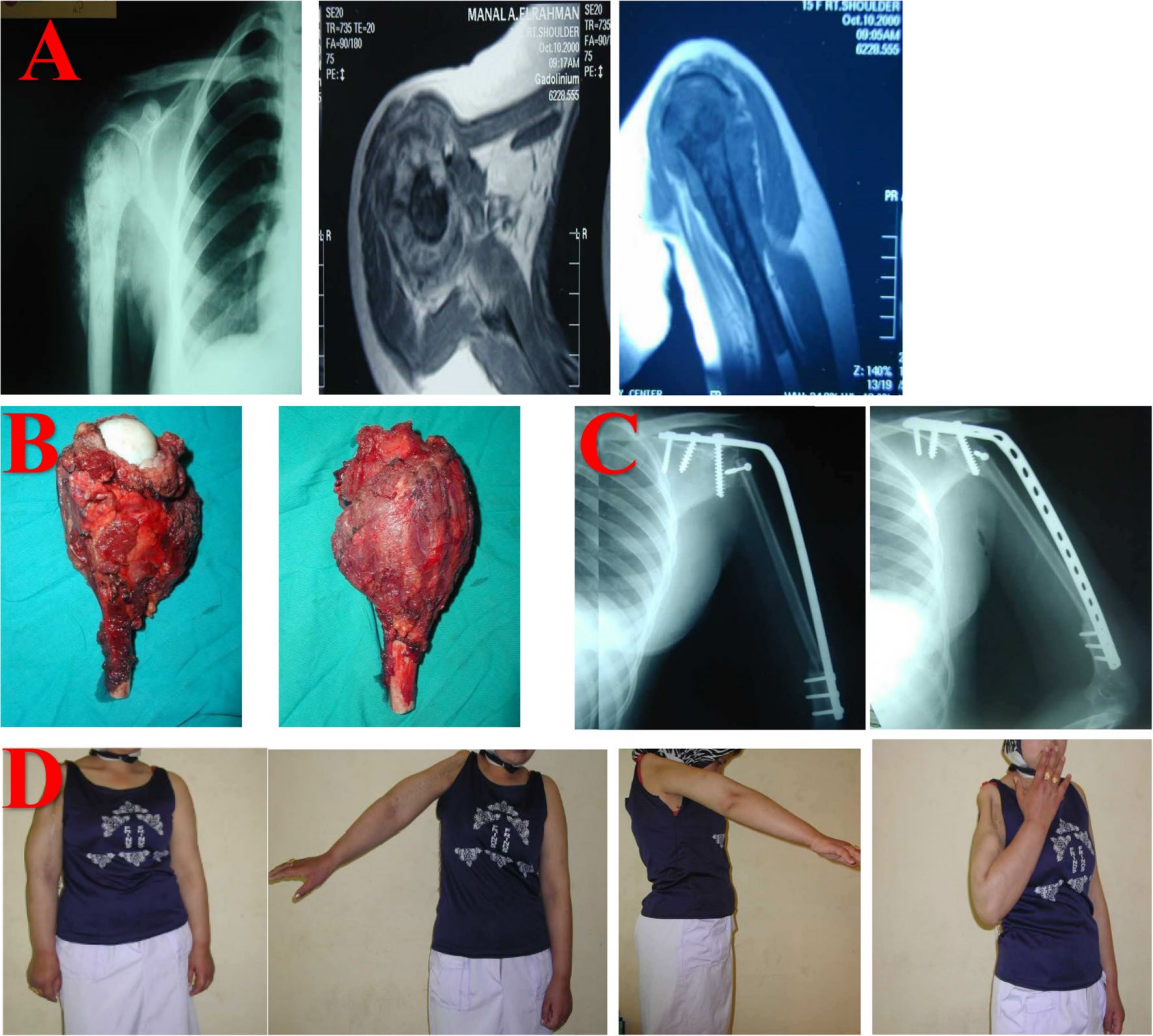
**A** Preoperative investigations.* Left*: X-ray of the Rt shoulder showed an extensively sclerotic lesion with a periosteal reaction, as indicated by a Codman triangle and sunburst appearance.* Center*: Axial-cut MRI of the shoulder with T1 gadolinium tumor enhancement.* Right*: Coronal-cut MRI demonstrating a high signal intensity on T2-weighted imaging. **B** Resected specimen. **C** Immediate postoperative X-ray (*left*) and imaging performed at 12 weeks of follow-up (*right*), with fusion at the humerus–implant junction readily visible. **D** At 6 months of follow-up, the patient showed satisfactory elbow flexion ROM (range of motion), varying between 10 and 120 degrees, while shoulder abduction was limited to 40 degrees

Our study was designed to assess the oncological outcome of proximal humerus osteosarcoma treated by limb salvage surgery and chemotherapy. Furthermore, we compared the functional outcomes after limb salvage using two main methods of reconstruction: biological reconstruction using vascularized grafts with shoulder fusion (Fig. 3) and nonbiological reconstruction using a PMMA spacer or endoprosthesis with a mobile shoulder. Finally, we assessed the prognostic factors that affect both the oncological and functional outcomes. We assessed oncological outcome in terms of 5-year survivorship, local recurrence and chest metastasis, while functional outcome was assessed using the MSTS score.

The 5-year survivorship in our cases was 65%, which is similar to that reported by Wittig et al. [17], who managed 23 patients with a proximal humerus resection for stage IIA and IIB together with an endoprosthesis. Yao et al. [18] estimated the 5-year survival rate at 71%. In 2009, Gupta et al. in 2009 reported on 23 cases, almost all of whom were treated by limb sparing, and noted that survivorship markedly decreased with time from 77% at 5 years of follow-up to 57% at 10 years [9].

Our study showed that local recurrence occurred in 18% of the patients and was not statistically correlated with any of the studied prognostic factors, especially resection length, deltoid resection, and pathological fractures on presentation. Our study obtained the same result as Gupta et al. [9] in terms of local recurrence, which occurred in 13% in their cases and was highly linked with positive margins.

Chest metastasis occurred in 41% of patients, which is higher than in Yao et al.’s study, where distant metastasis occurred in 30% [18].

In comparison with patients with osteosarcoma at other sites who were also managed in our institution, proximal humerus osteosarcoma patients showed higher incidence rates of local recurrence and chest metastasis along with lower 5-year patient and limb survivorships compared to distal femur, proximal tibia and proximal femur osteosarcoma patients (Table 5). This was also found by many other studies [12, 19].

**Table 5:** Comparison between the outcomes of osteosarcomas affecting different sites in our institution

Osteosarcoma site	Number of patients	Local recurrence (%)	Chest metastasis (%)	5-year survivorship (%)	Limb survivorship (%)	Construct survivorship (%)	MSTS score
Distal femur	82	3.7	14.6	95.6	98.8	67.7	26
Proximal tibia	55	5.5	18.2	83.6	88.2	82.4	26
Proximal femur	32	9.3	18.7	81	93	9	24
Proximal humerus	34	18	41	64.7	87.5	85.2	25

Therefore, we believe that osteosarcoma of the humerus has a slightly worse oncological outcome compared to osteosarcoma around the knee and a better outcome than spinal and pelvic osteosarcomas [7, 11]. This conclusion was also drawn in other studies, such as a South Korean study by Cho et al., who similarly identified a proximal humerus osteosarcoma as having a poorer survivorship compared to osteosarcomas at other anatomical extremities [6]. However, a review of 345 osteosarcoma cases was performed in 1975 by Campanacci et al., who observed that tumors affecting the proximal half of the femur and humerus had a poorer outcome [2].

The mean MSTS score of our patients was 25 (83%); the lowest score was 22 (73%), while the highest was 29 (96%). The functional outcome in our study was comparable to that reported by Wittig et al., who used an endoprosthesis for reconstruction and achieved an MSTS score of 80–90% [17]. Vitiello et al. also found an excellent functional outcome in a patient with proximal humerus chondrosarcoma managed by a wide resection and 3D-printed custom-made prosthesis [16].

In our study, both nonbiological reconstruction and biological reconstruction yielded the same mean MSTS score of 25; however, this comparison was not statistically significant (*P* = 1). This is because, whatever the reconstruction modality used, it ultimately acts as a hanger for the upper limb to preserve elbow and hand function. This is mainly due to the resection of the rotator cuff muscles (as well as the deltoid muscle in some cases). In cases with shoulder fusion, some range of motion of the shoulder is still present due to scapulothoracic movement. This overall lack of superiority of any reconstruction method over the other has been reported by several previous studies [13, 15, 18].

The functional outcome in our study was not affected by whether the resection length was less or more than 15 cm (proximal or distal to the deltoid insertion) (*P* = 0.444). However, in another study done to assess the functional outcome and shoulder instability of the reconstruction of proximal humerus metastases, El Motassime et al. found that patients with a resection length of > 10 cm had worse outcomes than those who had a resection length of 10 cm. They chose 10 cm as this was the minimum resection done considering the size of the smallest module of prosthesis used. However, in their study, the rotator cuff muscles and the deltoid were preserved in some cases, as they were unaffected by the tumor (metastasis); this was not the case in our study, which assessed only primary aggressive osteosarcoma [8].

The poorer functional outcome noticed in our younger patient group was also found in a study by Yao et al., who encountered constraints such as a small intramedullary canal, compliance with immobilization and poor soft-tissue coverage due to insufficient remaining adjacent tissues in this patient group [18].

Strong points in our study include a relatively large sample of exclusively proximal humerus osteosarcoma cases in a single center, with surgery and follow-up done by the same team. To the best of our knowledge, this is the highest cumulative number of cases of proximal humerus osteosarcoma currently studied. Another strong point of our study is the use of biological and nonbiological reconstruction modalities in almost all of our 34 patients, who showed similar functional outcomes to patients who had received an endoprosthesis elsewhere. The cost effectiveness without compromising the functional outcome should be considered as well in the management of proximal humerus osteosarcoma.

Our study encountered limitations that should be taken into consideration when interpreting the results, such as the combination of pediatric and adult populations, given that these groups require different reconstruction modalities, meaning different functional outcomes and different oncological outcomes. Also, being a retrospective study, it potentially allows selection bias; however, this can be justified by the rarity of the tumor at this site as well as the long follow-up required to evaluate the oncological outcome. Future systematic reviews and a meta-analysis analyzing the outcomes of proximal humeral osteosarcoma in particular are required to provide more solid data.

## Conclusion

Treatment of osteosarcoma proximal humerus by limb salvage and chemotherapy yields a good functional outcome. The method of reconstruction does not impact the resultant function. The 5-year survivorship of these patients is 65%. Younger patients have a better oncological outcome but an inferior functional outcome. Compared to other anatomical sites, osteosarcoma affecting the proximal humerus has a slightly worse oncological outcome.

## Data Availability

The datasets used and/or analyzed during the current study are available from the corresponding author on reasonable request.

## References

[1] Böhler C, Brönimann S, Kaider A, Puchner S, Sigmund I, Windhager R (2018). Surgical and functional outcome after endoprosthetic reconstruction in patients with osteosarcoma of the humerus. Sci Rep.

[2] Campanacci M, Cervellati G (1975). Osteosarcoma: a review of 345 cases. Ital J Orthop Traumatol.

[3] Chan YH (2003). Biostatistics 102: quantitative data–parametric & nonparametric tests. Singapore Med J.

[4] Chan YH (2003). Biostatistics 103: qualitative data—tests of independence. Singapore Med J.

[5] Chan YH (2003). Biostatistics 104: correlational analysis. Singapore Med J.

[6] Cho WH, Song WS, Jeon DG, Kong CB, Kim MS, Lee JA, Yoo JY, Kim JD, Lee SY (2010). Differential presentations, clinical courses, and survivals of osteosarcomas of the proximal humerus over other extremity locations. Ann Surg Oncol.

[7] Dekutoski MB, Clarke MJ, Rose P, Luzzati A, Rhines LD, Varga PP, Fisher CG, Chou D, Fehlings MG, Reynolds JJ, Williams R, Quraishi NA, Germscheid NM, Sciubba DM, Gokaslan ZL, Boriani S (2016). Osteosarcoma of the spine: prognostic variables for local recurrence and overall survival, a multicenter ambispective study. J Neurosurg Spine..

[8] El Motassime A, Meschini C, Di Costa D, Rovere G, Matrangolo MR, De Maio F, Farsetti P, Ziranu A, Maccauro G, Vitiello R (2023). Functional outcomes and shoulder instability in reconstruction of proximal humerus metastases. Curr Oncol.

[9] Gupta GR, Yasko AW, Lewis VO, Cannon CP, Raymond AK, Patel S (2009). Risk of local recurrence after deltoid-sparing resection for osteosarcoma of the proximal humerus. Cancer.

[10] Japie IM, Rădulescu D, Bădilă A, Papuc A, Ciobanu T, Stănculescu D (2020) Functional results of various reconstruction techniques in primary malignant bone tumors. Romanian J Orthop Surg Traumatol 3(1):15–19

[11] Kawai A, Huvos AG, Meyers PA, Healey JH (1998). Osteosarcoma of the pelvis. Oncologic results of 40 patients. Clin Orthop Relat Res.

[12] Li J, Wang Z, Ji C, Chen G, Liu D, Zhu H (2017). What are the oncologic and functional outcomes after joint salvage resections for juxtaarticular osteosarcoma about the knee?. Clin Orthop Relat Res..

[13] Liu T, Zhang Q, Guo X, Zhang X, Li Z, Li X (2014) Treatment and outcome of malignant bone tumors of the proximal humerus: biological versus endoprosthetic reconstruction. BMC Musculoskelet Disord 15(1):69 10.1186/1471-2474-15-69 PMC3975708 24607200

[14] Misaghi A, Goldin A, Awad M, Kulidjian AA (2018). Osteosarcoma: a comprehensive review. SICOT-J.

[15] Rafalla AA, Abdullah ESA (2017) Endoprosthetic replacement versus cement spacer in reconstruction of proximal humerus after tumor resection: Cost and benefits. J Orthop Surg Hong Kong 25(2):2309499017713937 10.1177/2309499017713937 28625098

[16] Vitiello R, Matrangolo MR, El Motassime A, Perna A, Cianni L, Maccauro G, Ziranu A (2022). Three-dimension-printed custom-made prosthetic reconstructions in bone tumors: a single center experience. Curr Oncol.

[17] Wittig JC, Bickels J, Kellar-Graney KL, Kim FH, Malawer MM (2002). Osteosarcoma of the proximal humerus: long-term results with limb-sparing surgery. Clin Orthop.

[18] Yao W, Cai Q, Wang J, Hou J (2020) Mid- to long-term effects of two different biological reconstruction techniques for the treatment of humerus osteosarcoma involving caput humeri. World J Surg Oncol 18(1):23 10.1186/s12957-020-1797-z PMC6990589 31996228

[19] Zhang C, Hu J, Zhu K, Cai T, Ma X (2018) Survival, complications and functional outcomes of cemented megaprostheses for high-grade osteosarcoma around the knee. Int Orthop 42(4):927–938 10.1007/s00264-018-3770-9 29427125

